# Altered Brain Activities Associated with Craving and Cue Reactivity in People with Internet Gaming Disorder: Evidence from the Comparison with Recreational Internet Game Users

**DOI:** 10.3389/fpsyg.2017.01150

**Published:** 2017-07-11

**Authors:** Lingxiao Wang, Lingdan Wu, Yifan Wang, Hui Li, Xiaoyue Liu, Xiaoxia Du, Guangheng Dong

**Affiliations:** ^1^Department of Psychology, Zhejiang Normal University Jinhua, China; ^2^CAS Key Laboratory of Behavioral Science, Institute of Psychology Beijing, China; ^3^Department of Psychology, University of Chinese Academy of Sciences Beijing, China; ^4^Department of Psychology, University of Konstanz Konstanz, Germany; ^5^Shanghai Key Laboratory of Magnetic Resonance, Department of Physics, East China Normal University Shanghai, China; ^6^Institute of Psychological and Brain Sciences, Zhejiang Normal University Jinhua, China

**Keywords:** recreational Internet game users, Internet gaming disorder, cue-reactivity, impulse inhibition, intense desire

## Abstract

Although the neural substrates of cue reactivity in Internet gaming disorder (IGD) have been examined in previous studies, most of these studies focused on the comparison between IGD subjects and healthy controls, which cannot exclude a potential effect of cue-familiarity. To overcome this limitation, the current study focuses on the comparison between IGD subjects and recreational Internet game users (RGU) who play online games recreationally but do not develop dependence. Data from 40 RGU and 30 IGD subjects were collected while they were performing an event-related cue reactivity task in the fMRI scanner. The results showed that the IGD subjects were associated with enhanced activation in the left orbitofrontal cortex (OFC) and decreased activation in the right anterior cingulate cortex (ACC), right precuneus, left precentral gyrus and right postcentral gyrus in comparison with the RGU subjects. OFC is involved in reward evaluation and ACC is implicated in executive control function based on previous researches. Moreover, the activation of OFC were correlated with the desire for game-playing. Thus, the higher activation in OFC might suggests high desire for game playing, and the lower activation in ACC might indicates impaired ability in inhibiting the urge to gaming-related stimuli in IGD subjects. Additionally, decreased activation in the precuneus, the precentral and postcentral gyrus may suggest the deficit in disentangling from game-playing stimuli. These findings explain why IGD subjects develop dependence on game-playing while RGU subjects can play online games recreationally and prevent the transition from voluntary game-playing to eventually IGD.

## Introduction

Internet gaming disorder (IGD), the most prevalent (57.5 percent) subtype of Internet addiction disorder (IAD) (Han et al., 2012; Ko et al., 2013; Chen et al., 2015), is defined as the incapacity to control the desire for obsessive online game playing, which leads to various functional impairments, such as social, financial, occupational, and behavioral difficulties (Young, 1998; Achab et al., 2011; Dong et al., 2012b,c, 2013b, 2014a; Ko, 2014; Ko et al., 2014; Petry et al., 2014). It has been regarded as a type of non-financial pathologic gambling (Griffiths, 2005; Han et al., 2012), a form of behavior addiction (Holden, 2001), or a type of impulse control disorder (Sadock and Sadock, 2007). Based on the similarities among IGD, substance disorder, and pathologic gambling, the DSM-5 proposed the diagnostic criteria for IGD in conditions for further study (American Psychiatric Association, 2013).

Craving is defined as the intense desire for the experience of a psychoactive substance or behavior (Blumenthal and Gold, 2009). It has been considered as the central feature of pathologic gambling and substance disorder (Ko et al., 2008). The degree of craving can be increased by addiction-related cues (Blumenthal and Gold, 2009), which is thought to play a critical role in developing and maintaining addictive behaviors (Carter and Tiffany, 1999; Tong et al., 2007; Goudriaan et al., 2010), as well as relapse to addictive behaviors (Cooney et al., 1997; Kosten et al., 2005; Marissen et al., 2006). Previous neuroimaging researches on substance dependence and pathological gambling have revealed abnormal brain activity in the orbitofrontal cortex (OFC), dorsolateral prefrontal cortex (DLPFC), anterior cingulate cortex (ACC), amygdala, hippocampus, and precuneus in response to addiction-relevant cues (Maas et al., 1998; Tremblay and Schultz, 1999; George et al., 2001; Wrase et al., 2002; Crockford et al., 2005; Filbey et al., 2008). Similarly, studies on the IGD have reported that compared to healthy controls (HC), subjects with IGD showed aberrant activation in OFC, DLPFC, ACC, precuneus, caudate nucleus in response to gaming pictures (Ko et al., 2008; Han et al., 2010; Sun et al., 2012; Liu et al., 2016; Zhang et al., 2016).

However, all these studies on the cue-reactivity of IGD focused on the contrast between IGD subjects and HC (Ko et al., 2008; Han et al., 2010; Sun et al., 2012; Liu et al., 2016). This method has some limitations. First, it failed to control the game familiarities between IGD and HC, as IGD subjects are more familiar with the gaming cues than the HC; Second, IGD subjects played online games a lot, however, the HC subjects are low-frequent/none game players, they have limited experience with online gaming. To overcome these limitations, it is important to include a specific group of game players—the recreational Internet game users (RGU) as the control group. RGU are individuals who play online games recreationally but do not develop transition to addiction (Viriyavejakul, 2008; Kuss and Griffiths, 2012). They do not show the core symptoms of addiction, such as loss of control, withdrawal, and conflict (Ko, 2014). More importantly, they do not meet the diagnostic criteria for IGD by DSM-5 and do not need treatment (Petry et al., 2014). Thus, the present study focused on the differences in neural activity of craving and cue-reactivity between IGD and RGU to expand the understanding of specific features of IGD, and explore risk factors and effective interventions for IGD.

As reviewed above, previous studies have demonstrated that IGD subjects reported stronger craving for game-playing and showed aberrant brain activities in regions responsible for reward evaluation, such as DLPFC, OFC (Ko et al., 2008; Han et al., 2010; Sun et al., 2012; Dong et al., 2017a) as compared to HC subjects. Accordingly, we expected similar brain activities to game relevant cues in IGD subjects as compared to RGU subjects. In addition, it has been noted that IGD subjects are associated with failures in controlling the desire for playing online games (Lin et al., 2015a). Numerous imaging studies have found impaired executive control ability in IGD subjects (Dong and Zhou, 2010; Dong et al., 2010, 2011b, 2012a, 2014b, 2015, 2017b; Dong and Potenza, 2014; Weinstein and Lejoyeux, 2015; Wang L. et al., 2016a,b; Wang Y. et al., 2016b; Weinstein et al., 2017), yet, the direct evidence for the impaired executive control ability in inhibiting craving for game-playing in the context of online gaming cues are still lacking (Ko et al., 2008, 2013; Han et al., 2010; Sun et al., 2012). Thus, the present study filled in the gap. We expected that IGD subjects would show dysfunctional brain activities in the executive-control-related regions.

## Materials and Methods

### Participants

The present study was approved by the Human Investigations Committee of Zhejiang Normal University. Forty RGU and 30 individuals with IGD were recruited in this study. All participants were right-handed and provided written informed content in accordance with the Declaration of Helsinki. Participants were screened according to their scores on Young’s online Internet addiction test (IAT) (Young, 2009), the nine-item diagnostic criteria of IGD proposed by the DSM-5 committee (Petry et al., 2014), and their weekly Internet gaming time. Young’s IAT consists of 20 items. Previous studies have testified the reliability and validity of IAT in classifying IAD (Widyanto and Mcmurran, 2004; Widyanto et al., 2011). Each item of Young’s IAT assesses the degree of Internet use-related problems (i.e., psychological dependence, withdrawal, and related problems in sleep, school, or work) on a 5-point-scale. Individuals who scored between 31 and 49 points are regarded as average online users who maintain control of Internet use, though sometimes they may spent a bit too long in surfing the Internet. Scores between 50 and 80 points reveal occasional or frequent Internet use-related problems as a result of uncontrolled Internet usage^1^.

Inclusion criteria for the IGD group were the following: (1) scored larger than 50 on Young’s IAT (Lin et al., 2015a,b; Wang L. et al., 2016a,b); (2) met at least 5 DSM-5 criteria; (3) play online games is their major Internet activity; (4) play online games more than 14 h per week, for a minimum of 2 years; (5) endorsement of League of Legends (a popular online game in China) as the only source of Internet online games. The inclusion of RGU is the key step of the current study. The inclusion criteria for the RGU group were used previously (Wang Y. et al., 2016a) and described briefly as follows: (1) scored less than 50 on Young’s IAT; (2) met fewer than 5 DSM-5 criteria; (3) play online games more than 14 h per week, for a minimum of 2 years; (4) endorsement of League of Legends as the only source of Internet online games; (5) reported no feeling of remorse or guilt about playing online games and stated that their regular use did not interfere with school, family, work, or social obligations. Exclusion criteria for all participants included (1) historical records of or current psychiatric/neurological disorders (e.g., depression, anxiety, schizophrenia and substance dependence) assessed by a structured psychiatric interviews (MINI) (Lecrubier et al., 1997); (2) previous or current use of gambling and illegal drugs (i.e., heroin, marijuana) or any other types of addictions (e.g., alcohol). Participants were required to not take any medicine or substances including tea and coffee on the day of scanning.

**Table 1** shows the demographic information of the two groups. There was no significant difference in age, BDI score, education level and the Internet gaming time between the IGD and RGU group, while the IAT scores and DSM-5 scores of the IGD group were significantly higher than those of the RGU group.

**Table 1:** Demographic information and group differences.

	IGD *N* = 30	RGU *N* = 40	*T*	*p*
Age (Mean ± SD)	21.07 ± 1.34	21.45 ± 1.32	-1.20	0.236
BDI score (Mean ± SD)	2.23 ± 0.82	2.05 ± 0.85	0.91	0.366
Years of education (Mean ± SD)	15.23 ± 2.33	15.78 ± 1.37	-1.14	0.262
IAT score (Mean ± SD)	65.30 ± 11.68	41.35 ± 10.19	9.14	0.000***
DSM-5 score (Mean ± SD)	5.80 ± 1.10	2.63 ± 1.37	10.42	0.000***
Years playing online games (Mean ± SD)	3.50 ± 1.07	3.34 ± 0.96	0.67	0.509
Game playing per week (Hours; Mean ± SD)	18.90 ± 9.13	20.13 ± 9.57	-0.97	0.334

*IGD, Internet gaming disorder; RGU, recreational Internet game users; BDI, Beck Depression Inventory; IAT, Internet addiction test; ^∗∗∗^*p* < 0.001.*

### Task and Procedure

**FIGURE 1:**
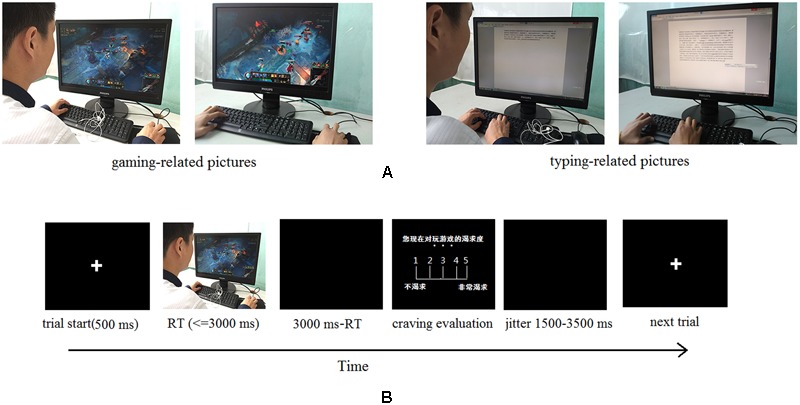
**(A)** Examples of gaming-related stimuli (left) and typing-related stimuli (right). **(B)** The timeline of one trial in the event-related cue-reactivity task. The test version of the task is in Chinese. “
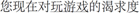
” means “The degree of your craving for game-playing right now”; “
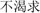
” means “no craving”; “
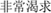
” means “extremely high craving.”

An event-related cue reactivity task was applied in this study. It contains two types of cue pictures: 30 gaming-related pictures and 30 typing-related pictures (neutral baseline). And in each type, half of the 30 pictures contained a face and the half contained a hand. As shown in **Figure 1A**, gaming-related pictures describe a person who is playing the online game (LOL) on a computer, with half pictures showing faces and the other half showing hands. In typing-related pictures, the same person is typing an article on keyboard in front of a computer. The task of participants was to answer whether there was a face in the picture. They had to press the button ‘1’ (refer to ‘yes’) on the keyboard when a face was present and press ‘2’ (refer to ‘no’) when there was no face presented.

**Figure 1B** shows the timeline of a sample trial in the task. Firstly, a fixed 500 ms of cross was presented, followed by a cue picture as stated above. All pictures were presented in a randomized order. Each picture was presented for up to 3000 ms, during which participants had to make a response. The screen turned to black after button pressing and lasted for (3000 – response time)ms. Then, in the craving evaluation stage, participants were asked to evaluate the level of their craving for the corresponding stimuli on a 5-point scale, 1 (no craving) to 5 (extremely high craving). This stage lasted for up to 3000 ms and was terminated by a button-press. Finally, a 1500–3500 ms blank screen was presented between each trial. The whole task contained 60 trials and took almost 9 min. The task was presented and behavioral data was collected by the E-prime software (Psychology Software Tools, Inc.). All participants were asked to fill out a 10-item gaming urge questionnaire, range from 1 to 10 to assess the gaming craving prior to the fMRI (functional magnetic resonance imaging) scan (Cox et al., 2001).

### Behavioral Data Analysis

The performance parameters for the cue reactivity task were mean reaction time (RT) and mean scores of craving (gaming-related minus typing-related), named as induced craving scores. In addition, the scores of craving prior to the fMRI scan, named as initial craving scores, were also analyzed. In order to examine the difference between the IGD and RGU group, we performed an independent sample *t*-test on these three parameters.

### Image Acquisition and Pre-processing

Functional MRI data were collected by a 3T MR system (Siemens Trio) with a gradient-echo EPI T2^∗^ sensitive pulse sequence in 33 slices, interleaved sequence, 3 mm thickness, 30 ms echo time (TE), 2000 ms repetition time (TR), 220 mm × 220 mm of field of view, 90° flip angle and 64 × 64 for matrix. All trials were presented using Invivo synchronous system (Invivo Company^2^) via a monitor in the head coil, which allowed participants to view the trials presented on the screen.

The fMRI data were analyzed using SPM8 (Statistical Parametric Mapping^3^). Images were sliced-timed, reoriented and realigned to the first volume. And then T1-co-registered volumes were normalized to an SPM T1 template and smoothed using a 6 mm FWHM Gaussian Kernel spatially. No participant was excluded due to large head motion coefficients based on the criteria (head motion < 2.5 mm and 2.5 degree).

### First-Level FMRI Analysis

In the present study, we applied a general linear model (GLM) to examine blood oxygen level dependence (BLOD) signal related to the two event types (gaming-related trials, typing-related trials) and others (missed or error response). GLM built a design matric to represent a combination of the experimental onsets convolved with a canonical haemodynamic response function (HRF), which included all trial conditions (gaming-related trials, typing-related trials, and missed trials) and six head motion parameters. Then, to improve the signal-to-noise ratio, a high pass filter (cut-off period was 128 s) was used to filter out low frequency noise.

### Second-Level Group FMRI Analysis

Second-level analysis was conducted at the group level. At first, we identified voxels that showed a main effect in the gaming-related trials versus the typing-related trials among each group (IGD, RGU). Secondly, we determined voxels that were significantly different in BOLD signal between the two groups [(IGD _gaming_ – IGD _typing_) – (RGU _gaming_ – RGU _typing_)]. We then identified clusters of contiguous significantly different voxels at an uncorrected threshold *p* < 0.005. Finally, these clusters were tested for cluster-level FWE (family-wise-error) correction *p* < 0.05. Specially, the AlphaSim estimation indicated that clusters extent of 15 adjoining voxels would achieve the FEW threshold *p* < 0.05 effectively. The smoothing kernel applied in simulating false-positive (noise) maps using AlphaSim software was 6.0 mm and was estimated from residual fields of the contrast maps being pooled into the one-sample *t-*test.

### Regression Analysis

To identify the correlation between brain activities and behavioral performances, we first extracted the BOLD signal from the mean value of the remaining clusters that showed between-group differences. Then the BOLD data for all subjects were submitted to robust regression analyses with the RT, the induced craving scores, the initial craving scores, and the IAT and DSM scores. Note, robust regression analysis was used here to eliminate the effect of outliers, which represents the correlations between brain activations and behavioral performances.

## Results

### Behavioral Performance

**FIGURE 2:**
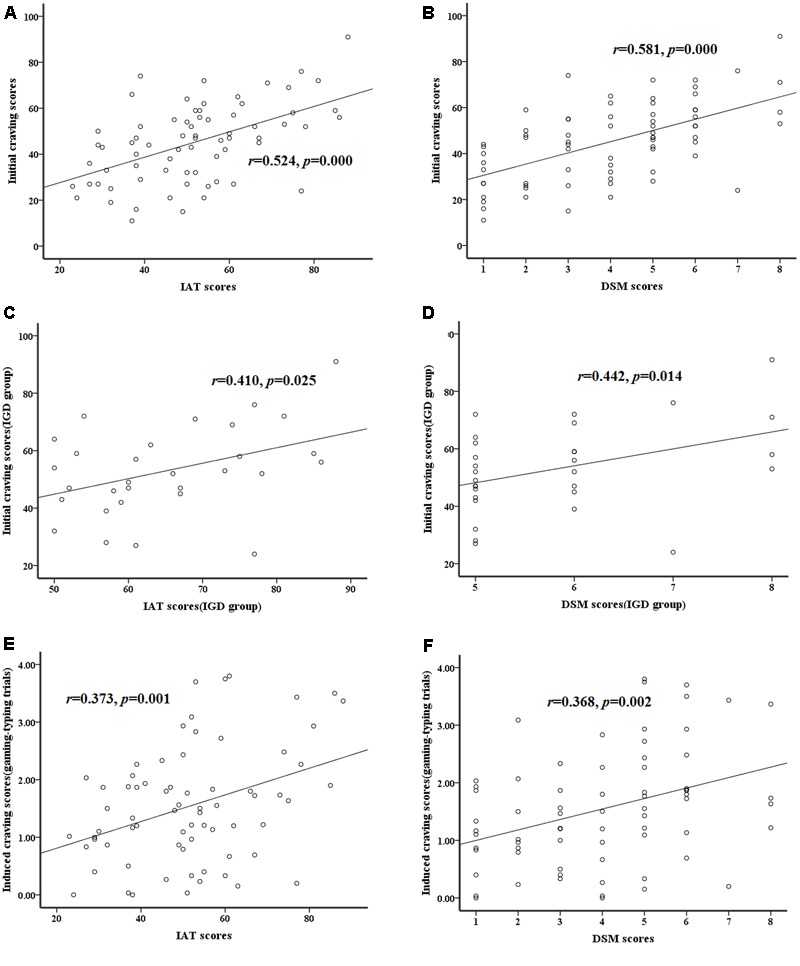
Correlations between the severity and the craving scores. **(A,B)** The initial craving scores show significantly positive correlation with the IAT scores, DSM scores for all subjects, respectively. **(C,D)** The initial craving scores show significantly positive correlation with the IAT scores, DSM scores for only IGD subjects, respectively. **(E,F)** The induced craving scores show significantly positive correlation with the IAT scores, DSM scores for all subjects, respectively.

The behavioral results showed significantly higher induced craving scores (IGD: 1.98 ± 1.10, RGU: 1.21 ± 0.78, *t*(1,69) = 3.25, *p* = 0.002) and initial craving scores (IGD: 53.10 ± 15.36, RGU: 39.13 ± 15.71, *t*(1,69) = 3.72, *p* = 0.000) in the IGD group as compared to the RGU group. No significant group-difference was found in the RT to cue pictures. Additionally, we found a significantly positive correlation between the IAT, DSM scores and the initial craving scores for all subjects (**Figures 2A,B**) and for the IGD group (**Figures 2C,D**). And the induced craving scores showed positively correlation with the IAT, DSM scores for all participants, respectively (**Figures 2E,F**).

### Imaging Results

**FIGURE 3:**
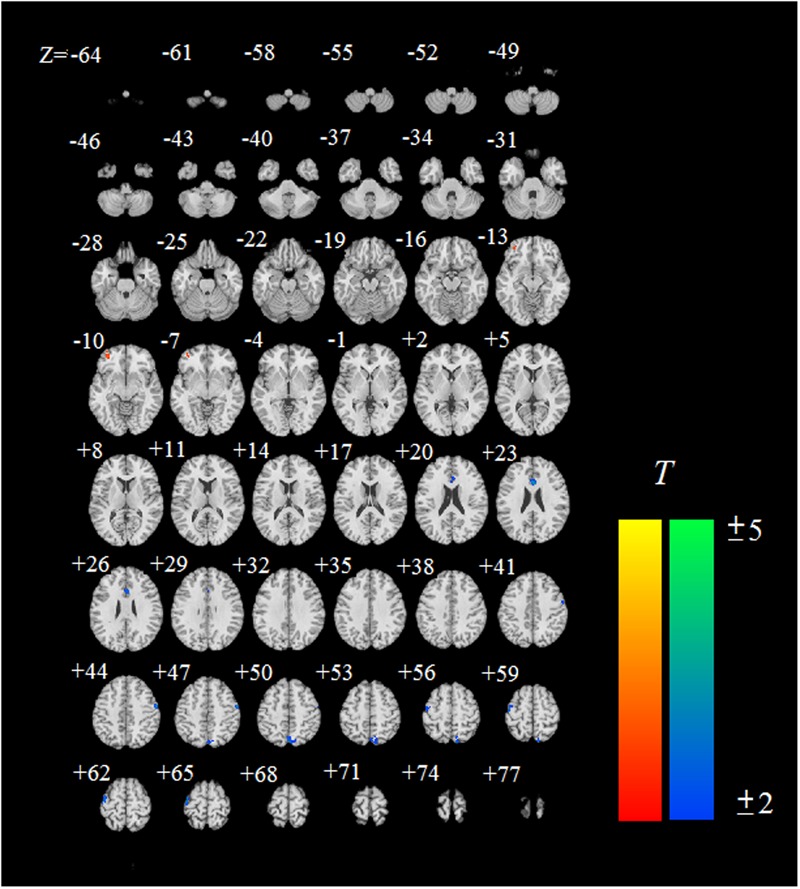
Brain regions showing significant difference in IGD subjects compared with RGU subjects. The IGD subjects showed enhanced activation (shown in red) in the left orbitofrontal cortex (OFC), and decreased activation (shown in blue) in the right anterior cingulate cortex (ACC), right precuneus, left precentral gyrus and right postcentral gyrus when comparing to the RGU group. ‘Z = ’ is the z coordinate of the slice in MNI space.

We examined the brain activities in the cue-reactivity task between the IGD and RGU group (**Figure 3** and **Table 2**). The IGD group showed increased BOLD signal activation in the left OFC compared to the RGU group, and decreased brain activities in the right ACC, right precuneus, left precentral gyrus and right postcentral gyrus in the IGD group when comparing to the RGU group.

**Table 2:** Brain regions showing significant group-difference in BOLD signal.

Regions	*x*	*y*	*z*	Size	BA	Max *t*
**IGD > RGU subjects**						
Orbitofrontal frontal gyrus (L)	-38	44	-9	17	11	3.54
**IGD < RGU subjects**						
Anterior cingulate cortex (R)	3	15	23	27	24	-4.14
Precuneus (R)	3	-75	43	34	7	-3.50
Precentral gyrus (L)	-40	-8	57	32	6	-3.76
Postcentral gyrus (R)	57	-12	42	15	3	-4.10

*We list significant clusters of increased (IGD > RGU subjects) or decreased (IGD < RGU subjects) activation to the gaming-related cues > typing-related cues. Shown are the coordinates of the local maxima in MNI space, the size of the clusters, the Brodmann Area, and the maximal *T*-statistic. Coordinates represent the local maxima in the gaming-related cues > typing-related cues contrast. BLOD, blood oxygen level dependence; IGD, Internet gaming disorder; RGU, recreational Internet gaming users; L, left; R, right.*

### Regression Analysis Results

**FIGURE 4:**
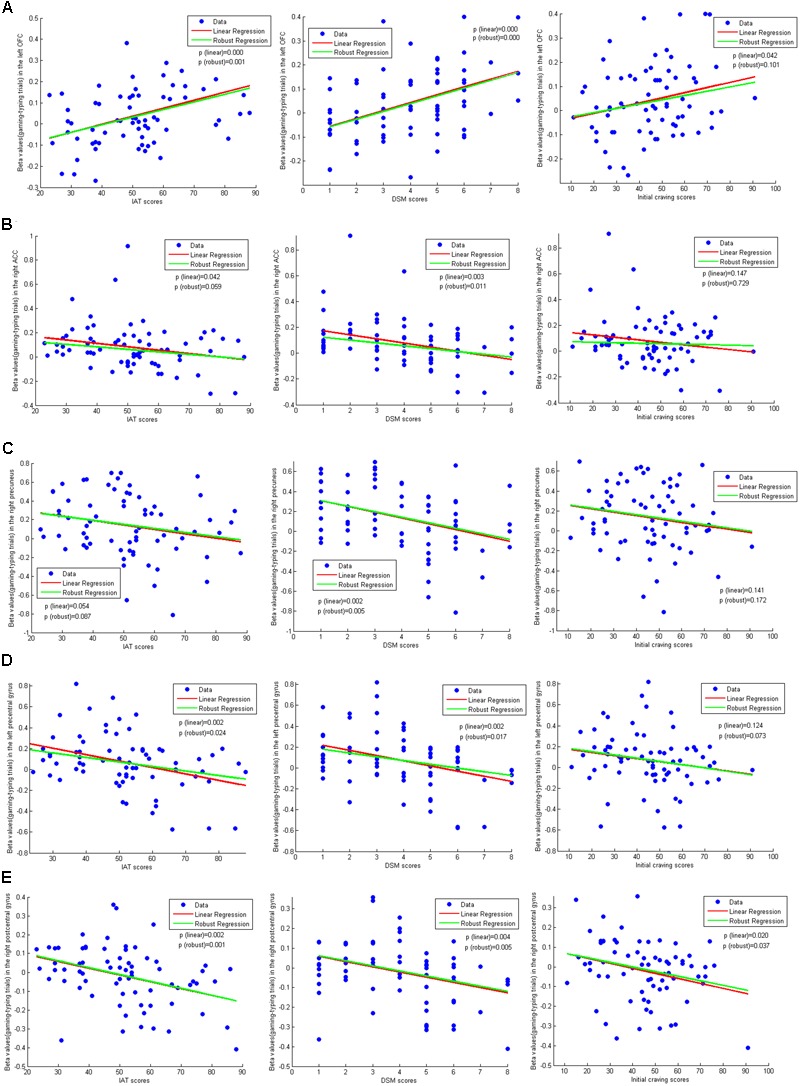
Regression relationship between brain activities and behavioral performances. p(linear) in each figure refers to the *p*-value of linear regression coefficient. p(robust) in each figure refers to the *p*-value of robust regression coefficient. **(A)** Shows the regression correlation between brain activation in the left OFC and the IAT scores, DSM scores and the initial craving scores, respectively. **(B)** Shows the regression correlation between brain activation in the right ACC and the IAT scores, DSM scores and the initial craving scores, respectively. **(C)** Shows the regression correlation between brain activation in the right precuneus and the IAT scores, DSM scores and the initial craving scores, respectively. **(D)** Shows the regression correlation between brain activation in the left precentral gyrus and the IAT scores, DSM scores and the initial craving scores, respectively. **(E)** Shows the regression correlation between brain activation in the right postcentral gyrus and the IAT scores, DSM scores and the initial craving scores, respectively.

As the robust regression lines in the **Figure 4**, there were significant regression correlations between brain activations in the OFC, ACC, precuneus, left precentral gyrus and right postcentral gyrus and the IAT, DSM scores, which means brain activations in the these regions were correlated positively or negatively with the IAT, DSM scores for all participants. At the same time, the regression correlations between brain activation in these regions (except ACC) and the initial craving scores were significant or marginally significant. Also, we put the results of linear regression in the figure to show the differences between linear regression and robust regression.

## Discussions

As far as we know, this is the first study comparing the neural activities associated with gaming-cues evoked craving between subjects with IGD and RGU. The IGD subjects reported higher scores of craving for game-playing and showed dysfunctional brain activation in the left OFC, the right ACC, right precuneus, left precentral gyrus and right postcentral gyrus as compared to the RGU group.

### Higher Desire for Game-Playing in IGD

The present imaging results demonstrated that the IGD subjects showed higher brain activity in the left OFC than the RGU group when exposed to the gaming-related cues. The OFC is widely thought to be involved in goal-directed behavior through assessing the significant stimuli and selecting appropriate behavior to achieve desired outcomes (Rolls, 2000). The similar feature has been reported in subjects with substance disorders, pathologic gambling, and online games addiction (Wrase et al., 2002; Myrick et al., 2004; Kosten et al., 2005; Franklin et al., 2007; Filbey et al., 2008). The OFC was found to be activated by expectations and delivery of reward (Elliott et al., 2000; Rolls, 2000; Schultz et al., 2000). It generates and maintains expectations for potential reward associated with reinforcement by integrating experiential history with current events (Bonson et al., 2002). These findings may reveal the important role of OFC in the craving for game-playing in IGD.

In current study, the IGD group reported significantly higher craving for online games than the RGU group both during and before fMRI scan. There was a positive association between the BOLD signal of the OFC, the initial craving scores, and the severity values of IGD (the IAT scores, DSM scores) among all participants. Thus, the larger the IGD values are, the stronger craving for gaming-playing and higher activation in the OFC would be observed. Taking all, we suggest that subjects with IGD generate expectations for game-playing through assessing the reward value of gaming behavior (which was induced by gaming-related cues) in the OFC. Therefore, they show stronger desire for game-playing than the RGU group, in line with the intense desire for drug-taking in drug addictions (Arthur et al., 2001; Goldstein and Volkow, 2002). Alternatively, the OFC might be involved in other functions, such as the inhibition and so on. Further studies are warranted to investigate this possibility.

### Impaired Control Ability in IGD

In the current study, decreased brain activity were detected in the right ACC in the IGD group comparing to the RGU group in response to gaming-related cues. Also, negative trends between the activities of ACC, the DSM and IAT scores were found among all participants, which suggest that the lower activation in the ACC is accompanied with higher severity of IGD. These results indicate that the ACC plays an essential role in the cue-reactivity of IGD, in accordance with prior researches on the cue-reactivity of IGD and other addictions (Ko et al., 2008; Goudriaan et al., 2010; Engelmann et al., 2012; Sun et al., 2012; Liu et al., 2016).

Convergent evidences have demonstrated that the ACC is involved in executive control function (Kerns et al., 2004; Dong et al., 2011a; Goldstein and Volkow, 2011; Wheelock et al., 2014). Executive control refers to one’s ability to direct or stop behaviors and thoughts, particularly when the behaviors (or thoughts) may not be advantageous or are regarded as improper (Goldstein and Volkow, 2011). Several neuroimaging researches have examined the impaired executive control ability indexed by dysfunction or structural abnormalities in ACC in people with IGD (Dong et al., 2011a, 2013a; Zhou et al., 2011; Wang et al., 2015; Dong and Potenza, 2016), as well as in drug addiction and pathologic gambling (Ruiter et al., 2011; Moeller et al., 2013; Santangelo et al., 2013; Schmidt et al., 2014; Feng et al., 2015). Better control ability in recreational online game users than online game addicts are associated with enhanced activation in ACC in a decision-making task (Wang Y. et al., 2016b). Combined with these findings, the present result may reveal a deficient executive control ability in IGD subjects, accompanied with comparably better control ability in RGU subjects. Additionally, subjects with IGD have been reported to link with deficit in executive control ability in cognitive tasks (Hester et al., 2010; Dong et al., 2011b; Wang et al., 2017) and are characterized by diminished control ability in Internet game-playing (Lin et al., 2015a). Besides, they showed higher scores in impulsivity (Lee et al., 2012) and thus are labeled as impulse control disorder (Sadock and Sadock, 2007). These behavioral phenomena of IGD are compatible with our results. Alternatively, the ACC is also implicated in attention processes (Bush et al., 1999; Seidman et al., 2006), thus, the lower activation in ACC might also suggest reduced attentional capacity in IGD subjects. However, considering the feature of the current event-related cue-reactivity task, which requires participants to suppress their strong craving for game-playing and focus on the task (pressing the correct button), and taking the findings above together, we speculated that IGD subjects show deficit in controlling their intensive desire for game-playing (provoked by gaming-related cues) as compared to RGU subjects, which is consistent with the impaired control ability to inhibit the craving for drug intake in drug addiction (Arthur et al., 2001; Goldstein and Volkow, 2002; Sinha and Li, 2007). Further studies are needed to investigate this issue. Notably, a cognitive-behavioral model of IGD proposed by Dong and Potenza (2014) have revealed enhanced desires for game-playing and poor control over such desires determined by the impairments of executive control function in individuals with IGD. The present result may contribute to certify the generalizability and availability of the model.

### The Roles of Precuneus, Precentral, and Postcentral Gyrus

Decreased brain activation in the right precuneus, left precentral and right postcentral gyrus were detected in subjects with IGD as compared to RGU subjects in response to gaming-related cues. In correlation analysis results, we found negative trends between the BOLD signal of the precuneus, the precentral, postcentral gyrus and the initial craving scores. Altered activities in these regions have been reported in previous studies about the cue-reactivity of IGD (Han et al., 2010; Sun et al., 2012; Liu et al., 2016). These results may indicate that the precuneus, the precentral and postcentral gyrus have a strong relationship with the cue reactivity in IGD.

A review about neural substrates of smoking cue reactivity has argued that the precuneus play an important role in cue reactivity (Engelmann et al., 2012). The precuneus has been proposed to contribute to attentional tracking of stimuli and the preparation of motor behaviors (Cavanna and Trimble, 2006). And it is involved in shifting attention between motor targets and motor imagery (Cavanna and Trimble, 2006). The precentral gyrus located in the Brodmann area 6 (pre-motor and supplementary motor cortex) is implicated in motor planning and execution. And the postcentral gyrus, as the primary somatosensory cortex, are the main sensory receptive region for the sense of touch. Besides, we found that the IGD subjects showed lower accuracy and longer RT than the RGU subjects, which demonstrated bad behavioral performance in subjects with IGD. On the aspect of our experimental task, participants were required to press buttons by observing if there was a face in the cue pictures with the background of game-playing. The lower activation in precuneus, precentral and postcentral gyrus in IGD may suggest the deficit in integrating visual and motor information from the cue-pictures and shifting attention from the stimuli of game-playing to the experimental task (pressing correct buttons). So far, the precuneus, precentral and postcentral gyrus received little attention in previous studies on the cue-reactivity of IGD. Thus, the present intriguing speculation may suggest that these three areas could be important areas of interest for further researches of cue reactivity in IGD.

### Limitations

There are some limitations of the present study to be noted. First, the causal relationship between IGD and the abnormal activity in the regions stated above could not be confirmed in the present study. It will be interesting to explore this relationship in future studies. Second, only seven female subjects were recruited for this study due to the higher popularity of online gaming in men than in women. Although they were balanced in the two groups (3 female IGD, 4 female RGU), the results may be biased. Further researches are needed to explore the gender effect in IGD. Third, several other variables, e.g., IQ, self-efficiency and socioeconomic status of the subjects, were not measured in the current study. The potential subgroup-difference in these variables may bias the results. Future studies should take these aspects into consideration. Finally, as the diagnostic criteria of IGD were still under consideration, the findings based on this criteria might be affected by it. A better diagnostic criterion of IGD might bring new insight into this issue.

## Conclusion

The present study examined different brain activation pattern between subjects with IGD and RGU using an event-related cue reactivity task. Hyperactive OFC indicates higher desire for game-playing and lower activation in ACC suggests impaired control ability in inhibiting the craving for game-playing in IGD subjects. Additionally, we infer that the decreased activation in the precuneus, precentral and postcentral gyrus may be associated with the difficulty in shifting attention from stimuli of game-playing to face-detection task in the IGD subjects. These findings explain why IGD subjects failed in preventing the transition from voluntary game-playing to eventual IGD, while as a contrast, RGU subjects can play online games recreationally without developing online-gaming dependence.

## Author Contributions

LxW analyzed the data and wrote the first draft of the manuscript. LxW, YW, HL, and XL contributed to experimental programming, data preprocessing. XD contributed to fMRI data collection. GD designed this research. GD and LdW revised and improved the manuscript. All authors contributed to and have approved the final manuscript.

## Conflict of Interest Statement

The authors declare that the research was conducted in the absence of any commercial or financial relationships that could be construed as a potential conflict of interest.

## Footnotes

1 http://netaddiction.com/internet-addiction-test/

2 http://www.invivocorp.com/

3 http://www.fil.ion.ucl.ac.uk/spm
